# Cystic Adventitial Disease of the Popliteal Artery With Recurrent Intermittent Claudication After Drug-Coating Balloon Angioplasty: A Case Report Treated by Surgical Treatment

**DOI:** 10.7759/cureus.23190

**Published:** 2022-03-15

**Authors:** Shinichi Tanaka, Kiyoshi Tanaka, Jin Okazaki

**Affiliations:** 1 Vascular Surgery, Kokura Memorial Hospital, Kitakyushu, JPN

**Keywords:** endovascular treatment, surgical treatment, popliteal artery, cystic adventitial disease, intermittent claudication

## Abstract

Cystic adventitial disease of the popliteal artery is a rare cause of unilateral intermittent claudication. The etiology of cystic adventitial disease is unknown, and affected patients are younger than those diagnosed with chronic arteriosclerosis. A 62-year-old man presented with a history of right leg claudication, which occurred after walking a distance of 500 m. The patient had no history of cardiovascular risk factors or trauma in the lower extremities. The ankle-brachial pressure index (ABI) was 0.58 in the affected leg. The patient was referred to the cardiovascular department. On Doppler ultrasonography, popliteal artery stenosis was detected. Following an angiogram, drug-coated balloon angioplasty was performed. The claudication improved, as indicated by an ABI of 1.11 in the affected leg. However, following one month of endovascular treatment, claudication had recurred, indicated by an ABI of 0.59. Computed tomography indicated the presence of a stenotic lesion in the popliteal artery, which may have developed from compression on the artery due to the presence of a surrounding periarterial cyst. The patient was subsequently diagnosed with cystic adventitial disease of the popliteal artery and was referred for vascular surgery. During surgery, the popliteal artery was exposed by the posterior approach; the artery showed circumferential enlargement and complete resection of the adventitial layer was performed. The patient had a successful postoperative recovery and the claudication disappeared (ABI of 1.14). Surgical management is an effective curative treatment for cystic adventitial disease of the popliteal artery that shows better efficacy than endovascular treatment. In the future, diagnostic methods for cystic adventitial disease should include computed tomography or magnetic resonance imaging with T1- and T2-weighted images.

## Introduction

Cystic adventitial disease of the popliteal artery leads to a rare cause of unilateral calf claudication [1]. Its etiology is currently unclear, and patients tend to be younger than those with conventional chronic arteriosclerosis [1]. Because of the likelihood of early recurrence of symptoms with endovascular treatment, surgical management is a more suitable curative treatment [2,3].

## Case presentation

A 62-year-old man presented with a history of right leg claudication and was admitted to our hospital. Claudication occurred following a 500-m walk. The patient had no history of cardiovascular risk factors, physical trauma, or extreme sports participation, and was a past smoker. The patient was initially referred to the cardiovascular department, presenting with a right ankle-brachial pressure index (ABI) of 0.70. Doppler ultrasonography and magnetic resonance angiography without T1- or T2-weighted imaging indicated stenosis of the right popliteal artery. Based on the imaging findings, the patient was diagnosed with chronic atherosclerosis obliterans and referred for endovascular treatment; however, an intravascular ultrasound indicated the possibility of external pressure. Therefore, cystic adventitial disease of the popliteal artery was suspected.

**Figure 1:**
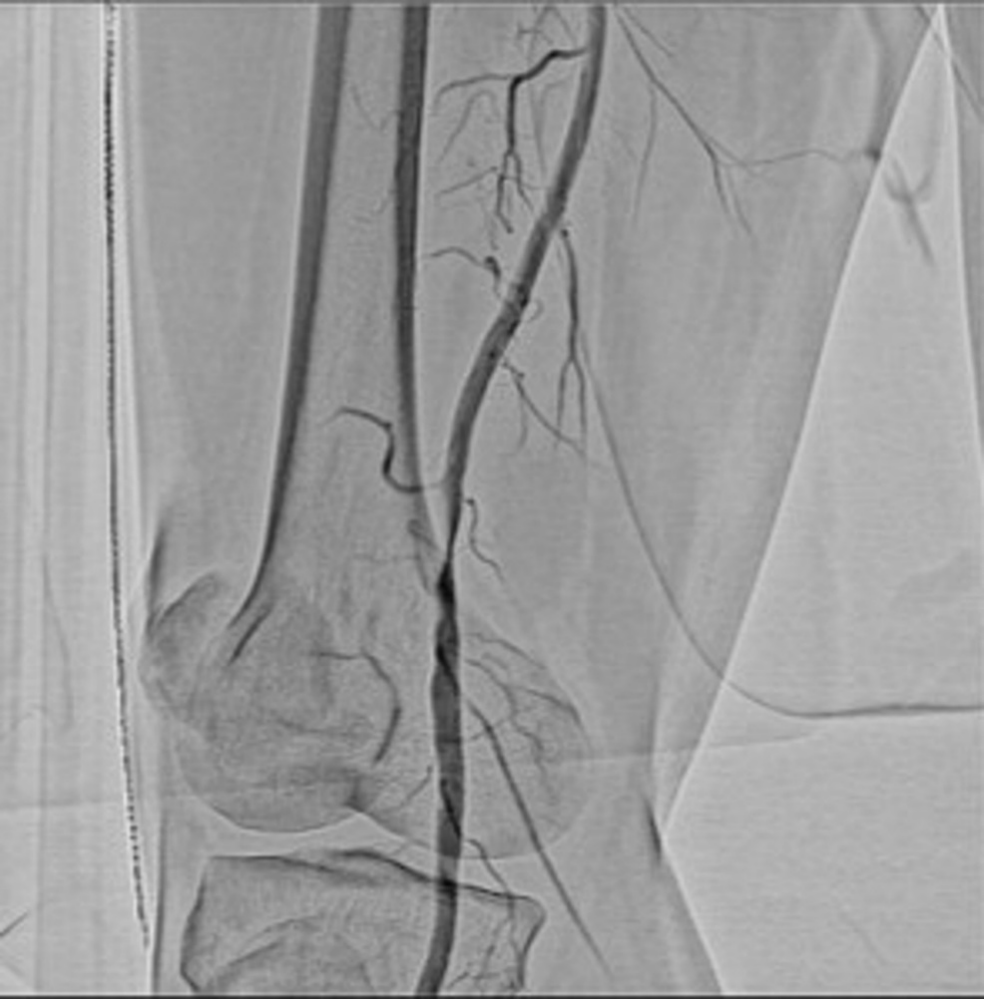
Angiogram image Angiogram of the patient of the popliteal artery before endovascular treatment.

**Figure 2:**
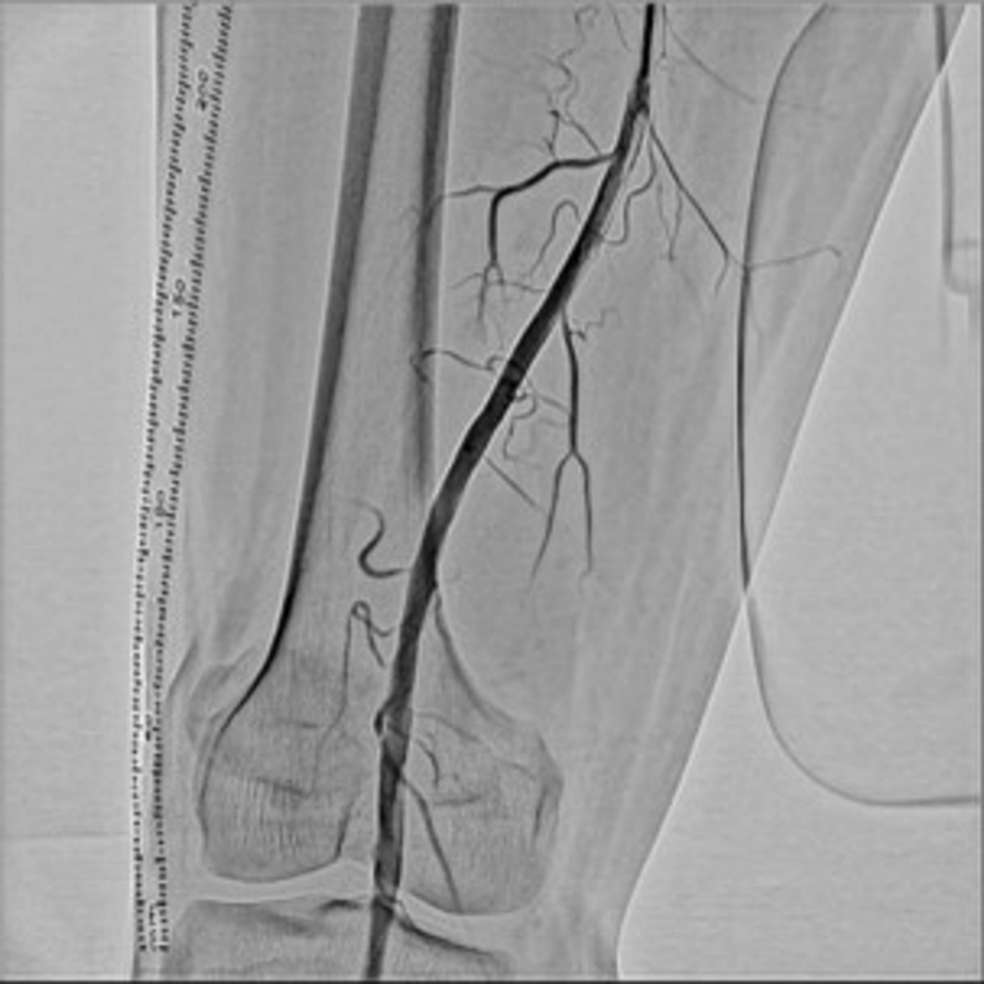
Completion angiography Angiogram image after angioplasty, indicating stenosis of the popliteal artery improvement.

Nevertheless, the patient underwent endovascular treatment. Drug-coated balloon angioplasty was performed and the stenosis of the popliteal artery was improved (Figures 1-2). Immediately following treatment, the claudication symptoms improved, and the ABI was recorded as 1.11 in the affected leg.

**Figure 3:**
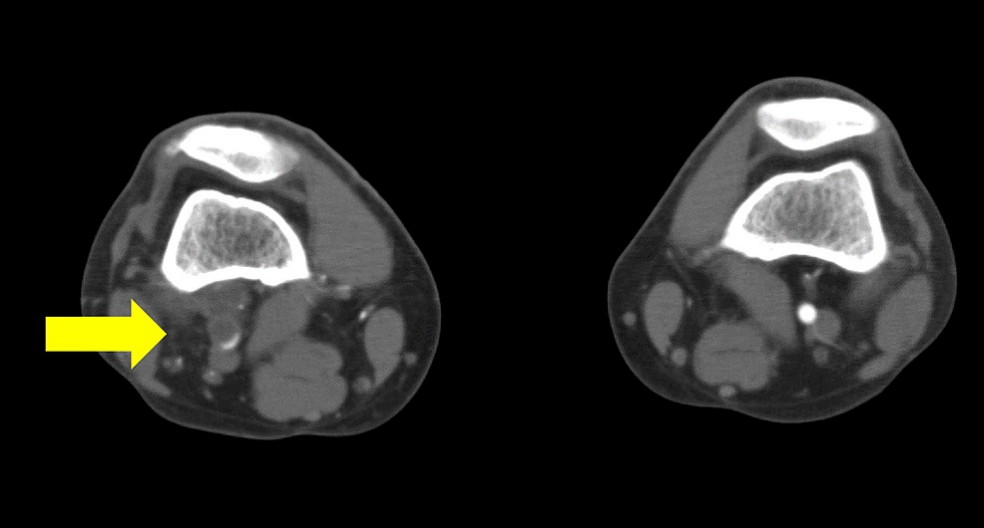
CT image CT image showing cystic adventitial disease of the popliteal artery. The arrow indicates an affected right popliteal artery, which is compressed by a non-enhancing structure surrounding the artery.

One month after endovascular treatment, the patient’s claudication had recurred, and the ABI of the affected side was 0.59. Therefore, the patient was examined further using enhanced computed tomography (CT). The CT findings indicated that the affected right popliteal artery was compressed by a non-enhancing structure that surrounded the arterial wall (Figure 3), and the patient was diagnosed with cystic adventitial disease of the popliteal artery and referred to our department.

**Figure 4:**
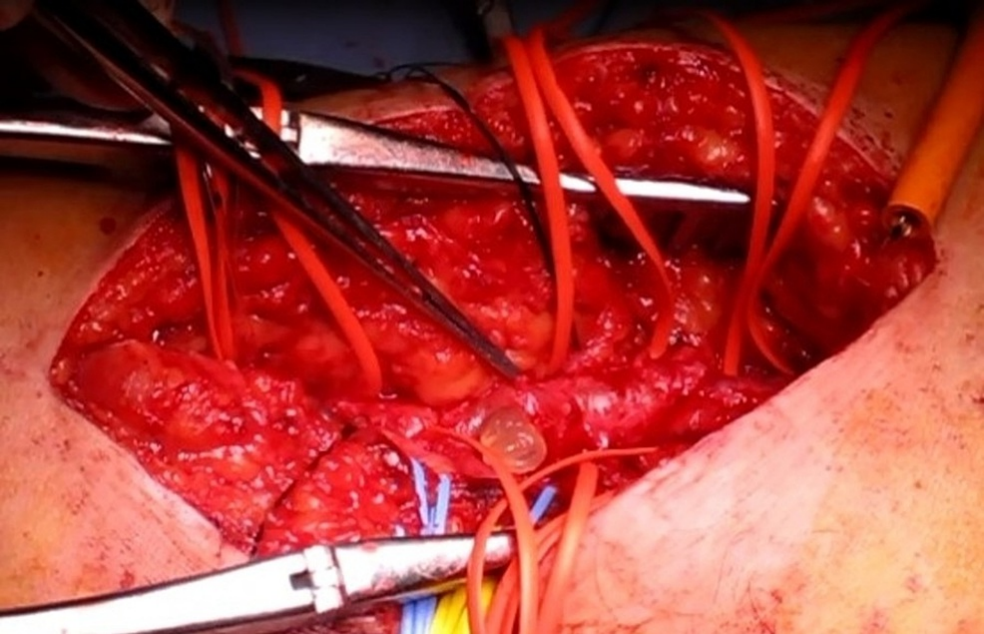
Intraoperative image Intraoperative findings showing popliteal artery and multiple surrounding cysts. The image shows a yellow, gelatinous substance leaking from the adventitial cyst.

Palpation disclosed no right popliteal or pedal pulse. A diagnosis of cystic adventitial disease was confirmed based on CT and angiography images. We performed surgery from a posterior approach, and a cystic enlargement on the anterior, lateral, and posterior sides of the right popliteal artery was observed. No connection between the cyst and the knee joint could be found. On incision, a yellow, gelatinous substance leaked from the multilocular cyst within the popliteal artery (Figure 4).

The affected adventitial layer was resected around the entire vessel wall. Following complete cystectomy, popliteal artery pulsation improved, and the anterior and posterior tibial arteries were palpated. Consistent with previous images, intraoperative ultrasonography did not show thrombus formation, and the media and intima of the artery at the cyst site were intact. Postoperative recovery was uneventful, and the final ABI measurement was 1.14. Following surgical treatment, the patient did not complain of any recurrence of claudication or discomfort in the affected leg.

## Discussion

Cystic adventitial disease is a non-atherosclerotic disease because of a functional occlusion of an artery or vein caused by compression by a cyst within the adventitial wall [4]. The disease often occurs unilaterally and most commonly affects the popliteal artery [1]. This disease affects males predominantly, with a male to female ratio of 15:1, and the representative patient age is in their mid-40s [4,5]. The disease accounts for 0.1% of lower-extremity claudication in patients who do not have atherosclerotic risk factors [6]. CT and magnetic resonance imaging with T1- and T2-weighted imaging have been recommended for use in the diagnosis of cystic adventitial disease of the popliteal artery [4], as they are more beneficial for the evaluation of cyst morphology and recognizing the cyst’s relationship to surrounding structures, for example, an adjacent joint. Moreover, these imaging methods prevent cyst enhancement with contrast administration. Doppler ultrasonography is a less invasive and more convenient diagnostic modality compared with other imaging methods. However, this modality is sometimes misleading and causes misdiagnosis, as demonstrated in the present case.

Cystic adventitial disease often causes restenosis in the short term, even following endovascular treatment, as in the present case, and its outcomes are poor [2,7]. A literature search (1980-2021, PubMed) shows only one report of successful endovascular treatments to date [8]. The report describes two cases of primary arterial stenting for popliteal adventitial cystic disease and persistent 4- and 10-year symptomatic alleviation and arterial patency [8]. The authors state that the secret to successful treatment is that the radial force of the bare-metal self-expanding stent pushes the adventitial cyst apart and dilates the lumen [8]. There have been no reports of cases of performing endovascular treatments using a drug-coated balloon. The cyst is underneath the adventitia, so it is natural to expect balloon angioplasty to fail. Furthermore, otherwise undergoing bad change, the healthy intima of the artery can be injured during endovascular treatment, inducing a higher risk of arterial thrombosis [9]. Recently, cases have been reported in which the cyst of the popliteal artery spontaneously regressed [10]. However, surgical management is the standard treatment for this disease [11]. Cystic excision and autologous venous graft interposition or bypass are the two main methods of surgical treatment. It is considered that not all cases require revascularization, such as interposition by grafting or bypass. Good long-term results have been reported with only the complete excision of cysts in cases without intimal damage [12]. As reported, because the border between the cystic lesion and the tunica media is visible, complete resection of the cyst from the arterial wall can be performed by just dissecting along the wall of the cyst [12]. In our case, no arterial damage was apparent on operative ultrasonography, so the patient did not need arterial reconstruction and underwent a complete cystic excision. A higher incidence of treatment failure or recurrence has been reported for complete cystic excision without arterial reconstruction (10-34%) than for revascularization surgery (0-10%) [11,12,13]. However, in several reports, results are poorly described, including a lack of follow-up. Therefore, accurate incidences of recurrence post each procedure cannot be given [14].

We must emphasize throughout the present case that it is important to make an accurate diagnosis of this rare vascular disease. In cases of intermittent claudication in younger patients, care is required to differentiate between cystic adventitial disease and conventional chronic arteriosclerosis obliterans. For an accurate diagnosis, enhanced CT or magnetic resonance imaging with T1- and T2-weighted images should be performed in addition to Doppler ultrasonography.

## Conclusions

Here, we report a case of cystic adventitial disease of the popliteal artery with recurrent intermittent claudication after paclitaxel-coated balloon angioplasty. The patient was successfully treated with surgical cyst excision. In the future, the treatment of intermittent claudication in younger patients should be approached with care to ensure appropriate diagnosis, and diagnostic methods should include CT or magnetic resonance imaging with T1- and T2-weighted imaging.
